# Fostering students’ creative thinking skills by means of a one-year creativity training program

**DOI:** 10.1371/journal.pone.0229773

**Published:** 2020-03-20

**Authors:** Simone M. Ritter, Xiaojing Gu, Maurice Crijns, Peter Biekens

**Affiliations:** 1 Institute for Management Research, Nijmegen School of Management, Radboud University, Nijmegen, The Netherlands; 2 Behavioural Science Institute, Radboud University, Nijmegen, The Netherlands; 3 Brainnovation Foundation, Eindhoven, The Netherlands; 4 Fontys University of Applied Sciences, Venlo, The Netherlands; Southwest University, CHINA

## Abstract

Creative thinking is among the most sought-after life and work skills in the 21^st^ century. The demand for creativity, however, exceeds the degree to which it is available and developed. The current project aimed to test the effectiveness of a one-year creativity training program for higher education. The creativity of students following the training was measured before, halfway, and after the training. In addition to the within-subjects comparison across time, performance was compared to a matched control group. At each of the measurement points, different versions of seven well-validated creativity tasks (capturing divergent and convergent creative thinking skills) were employed. The creativity training increased students’ ideation skills and, more importantly their cognitive flexibility. However, no difference in originality was observed. Finally, an increase in performance was observed for one of the convergent creativity tasks, the Remote Associate Test. Implications for educational settings and directions for future research are discussed.

## Introduction

From the first wheel to the latest microprocessor creativity has continuously enriched our lives. It plays a vital role in science, innovation, and the arts [1–3]. Moreover, the significance of creativity has also been recognized in daily life problem solving [4], in maintaining and fostering our well-being [5], and in successful adaptation to change [4, 6]. Creativity—the ability to generate original and useful ideas [7–9]—drives us forward, and it is among the most sought-after life and work skills in our complex, fast-changing world.

We have moved from an Industrial Age, to a Knowledge Age, to an Innovation Age. Many jobs are disappearing, and new jobs are emerging, for example, due to the transformative impact of digital technologies. On average our future generation of employees will change jobs more than 10 times before they reach the age of 50 [10]. As we don’t know how the future work field will look like, it is difficult to predict for what kind of jobs we have to prepare our current generation pupils and students. Whereas for decades content knowledge was a prerequisite for work, in the era of google we need individuals who are capable to creatively *use* and *generate* knowledge. To remain competitive, nations, organizations and individuals have to be able to think differently and to make connections between seemingly unrelated things. Global surveys have revealed that organizational leaders are mostly satisfied with their employees’ content knowledge or technical skills [11]. However, what they complain about is the lack of creativity in many otherwise qualified graduates [11]. For example, as reported by a UK employment survey, information technology graduates fail to grasp job opportunities due to a lack of creativity [12]. Creativity is not anymore, a ‘nice to have’, but has turned into a ‘must have’. Interestingly, the majority of employees indicate that they wish they had more creative ability (75%), and that they lacked exposure to creative thinking during their education (82%; [13]). Supporting these findings, recruiters denoted that creative thinking is a skill that is hard to find in job applicants [14]. All in all, the demand for creativity exceeds the degree to which it is available at all levels of the system. To meet the needs of the 21st century, academics, business leaders, and policy makers around the world have stressed that creativity should be fostered in the entire population [15].

Evolution has equipped us with a creative mind. However, we often do not use our creative thinking skills to the best of our ability. Some scholars even state that the educational system diminishes our creativity. In the most watched TED talk of all time, educationalist Ken Robinson claims that schools kill creativity—schools do not foster growing into but out of creativity. This is a rather radical view, as schools cultivate the knowledge on which creativity often depends. In schools, children develop the literacy skills necessary for all further learning. Creativity does not happen in a vacuum, it is based on knowledge. However, what schools mostly don’t focus on is teaching and practicing how existing knowledge can be used to come up with creative ideas and problem solutions. In schools that focus on creativity, it is often observed that creativity development is embedded in arts subjects, but not in subjects such as writing and mathematics [16]. Cotter, Pretz and Kaufman [17] studied the relationship between university applicants’ creativity, extracurricular involvement and traditional admission criteria (e.g., SAT scores, high school rank). The results revealed that applicants’ extracurricular activities positively predicted their creativity, whereas their academic performance or the traditional admission criteria even showed a negative relationship with creativity.

Creativity is a mental phenomenon that results from the application of ordinary cognitive processes such as working memory, and the ability to categorize and manipulate objects (creative cognition approach; [18, 19]). Importantly, the ability to think creatively can be taught and developed—creativity is not a fixed inborn trait [20–23]. However, this is often not what is happening in education. While the world has gone through revolutionary changes, teaching practices have not changed much. The main focus in education is still on rote learning. In classroom activities as well as in the curricula, little attention is paid on introducing and practicing cognitive strategies proven to foster creative thinking skills.

By now, a variety of reports stress that creative thinking is a crucial 21^st^ century skill [24–26], and a skill that should be fostered in schools [9, 27]. Schools allow not only the training of a creative elite, but of our entire future generation. To illustrate, simply the way a question is asked can either stimulate or undermine creative thinking: Example ‘What is three plus three?’ requires *convergent* thinking (i.e., finding the single, correct answer). However, if the teacher instead asks ‘Which calculation will result in six’, *divergent* thinking is stimulated—after all, the answer could be three plus three, two plus four, or twelve divided by two, and infinitely many others. Instead of focusing on calculations, the teacher could also ask a broader question: ‘What is six?’ The answer might be a triangular pyramid, the sixth sense, or an ice crystal. To boost creativity further, the teacher may ask ‘What can you do with six?’ Next day, she asks for answers. A dreamer or gifted visionary may answer: I see an array of hexagons, which you can use to build spaces. This example demonstrates that creativity is a skill that can be taught and developed within different academic domains and school subjects [28]. We can think of the brain as a muscle. To run a couple of kilometres, people must practice. By exercising regularly, our muscles and condition become strong enough to run a longer distance. It is no different for the brain. Regular exercise is required to develop a creative thinking style and to keep our brain in shape. A potentially helpful framework for fostering creativity in educational settings is the 4 P’s model of creativity: how to promote the cognitive processes that lead to creativity (Process), how to recognize and support creative individuals (Person), how the school/classroom environment impacts creativity (Press), and how to recognize and evaluate creativity in students’ work (Product).

### The current project

During recent years notable efforts have been made to empower creativity in education [29–31]. However, empirical evidence on the effectiveness of creativity intervention programs is often lacking. As concluded by Davies and colleagues [32] in their review paper, “Much literature in this area tends to be either philosophical, anecdotal or polemical, which has led to a strong belief about the effectiveness but significant evidence gaps” (p.89). To fill this gap, the current study aimed to develop and scientifically test the effectiveness of a creativity training program. The main objective of the current project was to scientifically test the effectiveness of a recently developed one-year creativity training program for higher education, called the ‘Brainnovation Six Step Cycle of Creativity’. The training had to fulfil several requirements: First, it has to be suitable for students with various educational backgrounds (i.e., it has to be domain unspecific). Second, it applies a cognitive approach, as previous research has shown that cognitive-oriented training programs have larger effects [20]. Third, it has to combine scientific insight and practical experience. Brainnovation is based on linking practical experience and anecdotal evidence (e.g., sleeping on a problem, distraction, connecting seemingly unrelated things) with existing models of the creative process (e.g., preparation, incubation, illumination and verification [33]) and with brain science (e.g., the finding that creative thinking is related to the interaction of three major brain networks; the central executive network, salience network and the default mode network [34–36]. The core of the Brainnovation method is the ‘Six Step Cycle of Creativity’. The first three steps explore the resources of the central executive network, and the last three steps explore those of the default mode network. A set of assignments trains the fluent application of all six steps. The idea is that by following the training, the student can apply the Six Step Cycle of Creativity to problems that need a creative solution. Four tools are employed to facilitate walking through and practicing the Six Step Cycle. The Six Step Cycle and the four tools are described in more detail in the Method section of the current paper. Fourth, rigorous scientific testing of the effectiveness of the training has to be performed: The creativity of students following the creativity training was measured before the training, halfway the training, and after the training. In addition to the within-subjects comparison across time, the creativity of students following the training was compared to a matched control group. At each of the three measurement points, seven well-validated creativity tasks were employed to test participants’ divergent thinking, convergent thinking and creative problem solving ability. The creativity measurement tasks are described in more detail in the Method section of the current paper.

We formulated the following hypotheses:

1. There will be a significant improvement in students’ creative thinking skills from pre-measure to half-way and post-measure in the training group. For exploratory reasons, we will also compare creative performance in the creativity training group between the half-way measure and the post-measure, as this gives an indication whether the time duration of the training has a positive effect on students’ creativity development.
2. In the control group no difference in creative performance is observed across the three measurement times.
3. The training group significantly differs in creative performance from the control group on the half-way measure and on the post-measure.

## Method

### Participants

The current study was conducted from September 2017 to May 2018 at an applied university in the Netherlands. The study was pre-registered on open science framework (see https://osf.io/znw5h/register/5730e99a9ad5a102c5745a8a). An a priori power analysis using G* power [37] was calculated. To reach a statistical power of .80, 215 students should be recruited for the study. The total participant number is slightly lower (198 instead of 215), as less than expected freshmen students enrolled in the program in the study year 2017/18. From the 198 students, 133 students followed the creativity training, a 5 ECTS (i.e., 140 hours) course entitled ‘Applied Creativity’. Another 65 students, who were not enrolled in the course, formed the control group. The training and the control group are comparable in terms of educational level (all freshmen) and educational background (Business related study). As preregistered, participants who did not regularly (less than 2/3 of all lessons) attend the creativity training program were excluded from data analyses. From the 78 participants who met this criterion, 57 were in the creativity training group, and 21 in the control group. 27 of the 78 participants were female and 50 were male, and the average age was 19.72 (*SD* = 1.82), ranging from 18 to 26 years. The study was conducted according to the principles expressed in the Declarations of Helsinki. The research was not of a medical nature, no minors or persons with disability were involved, and there were no potential risks to the participants; therefore, ethical approval was, when data collection started, not required by the Institution’s guidelines and national regulations. Importantly a lecturer prior to the study assigned each participant a subject identification code that was used in the current study. This code was not shared with the researchers, to make sure that personal data is staying within the educational institution.

### Procedure

The study employed a pre-post-test between-subject design. Participants were either in the creativity training group or in the control group. Participants’ creative thinking skills were assessed at three time points: at the beginning of the training program (pre-measure; beginning of the academic year, September 2017), after three months of the training (half-way measure; December 2017), and at the end of the training program (post-measure; May 2018). At each testing session, participants’ creative performance was measured by means of seven well-validated and frequently used creativity tasks (for tasks and task description, see the creativity measurement section).

### Creativity training

The creativity training program is provided as a mandatory course that counts for 5 ECTS credits. According to Dutch law, 1 credit represents 28 hours of work, and 60 credits represents one year of full-time study. The creativity course (in total 140 hours) lasted two semesters, and the course entailed lectures (i.e., focus on theory) and factory lessons (i.e., focuses on practice exercises in the field of international business).

In the creativity training program, students learned to apply the Six Step Cycle of Creativity to a wide range of problems. The 6 steps—understanding the question, convergent thinking, divergent thinking, detached thinking, stop thinking, and sleeping—are described in more detail below.

*Understand the question*. The problem must be defined correctly; failing to do so interferes with the other steps of the creative cycle [38]. This step requires a high focus. *Convergent thinking*. Convergent thinking is logic reasoning, straightforward thinking from A to B. People in general are quite good in convergent thinking, as schools put heavy focus on convergent thinking. *Divergent thinking*. Divergent thinking is associating freely without criticizing ideas or thoughts: One tries to consider different kinds of alternatives. To illustrate these steps with an example: The question ‘What is three plus three?’ elicits convergent thinking—there is one single correct answer, six; whereas the question ‘What is six?’ stimulates divergent thinking, it could be three plus three, nine minus three, and infinitely many other options. *Detached thinking*. In this stage, one tries to look at a problem with defocused attention [39] and without emotions or personal concern [40]. One can observe a problem, object, or image from all sides, upside down, turn it around, and toss and touch it. Central in this stage is a playful mood or a meditative mind set [41]. When answering the question ‘What is six?’ with detached thinking, the answer might be a triangular pyramid, a dice, an ice crystal, a hexagon, and so on. *Stop thinking*. If convergent, divergent and detached thinking did not provide a solution, a possible avenue may be to stop thinking about the problem. Let the problem ‘go’ for a while, create an incubation period [42, 43], for example, go shopping, go for a run, watch TV, dance, bike, bath, shower, drive, or listen to music [44]. Without conscious awareness, the unconscious is working hard [45] to re-assemble the information obtained in the previous steps of the cycle in new networks. Suddenly, an idea may pop-up, and experience that is described as an Euraka moment—often experienced at times when people expect it the least [46]. In terms of our example, the question ‘What is six?’, stop thinking may result in abstract, remote associations and the answer may be the Six Thinking Hats, or the sixth sense. *Sleeping*. A very powerful step of the cycle is sleeping on it. Research has shown a positive relationship between creativity and sleep [47, 48]. This step starts with deliberatively re-activating the problem just before going to sleep; this gives guidance to the unconscious where to focus on during sleep. It has, for example, been shown then even in rats [49] during sleep the unconscious starts to replay many scenarios to find a solution to a given problem [50, 51]. During sleep, the brain takes into account different kind of scenarios. An important advantage of the unconscious mind is that it is not hindered by social conventions or prejudices, and in that setting, mood and other variables are changing at an incredible speed, thereby allowing a diversity of option to be explored. This process may take one or more nights, and eventually it may lead to a creative idea. It may be helpful to put a notebook next to the bed. In case one wakes up in the night with a hunch, one must write it down, as people often do not remember their dreams and solutions the next morning [52]. For example, sleeping on the question ‘What is six?’, an architect may dream of floating images of hexagons, which in the case of Li Hu of the Beijing-based Open Architecture office, resulted in the design of the HEX-SYS—a reconfigurable construction system of hexagons in response to the proliferation of temporary structures erected by property developers during China’s recent construction boom.

Four tools are provided to facilitate walking through the Six Step Cycle: simplify (i.e., reduce the complexity of questions), differentiate (i.e., wonder what is more and less important; what is the big picture what are details), visualize (i.e., use real objects, make sketches, or imagine comparable processes from everyday life) and tag the problem (i.e., link the problem to one of the five senses: sight, smell, sound, taste, touch). Students are repeatedly provided with four different types of assignments, which trigger them to practice the different steps of the Six Step Cycle: The *Detox assignments* are provided at the start of the course, and they aim to train the flexibility of the mind by questioning prejudices and by fostering an open mind. The *Training assignments* focus on the first three steps of the Six Step Cycle, which train students’ cognitive creativity and their ability to quickly form remote associations. The *Jump assignments* are complex problems that are not easy to solve, and they challenge the students to employ and practice step four to six of the Six Step Cycle. Whereas the Training assignments must be solved within a short amount of time, for the Jump assignments students have one week, allowing unconscious processes to come into play. Often the Six Step Cycle must be repeated to find a solution. In addition to the three assignments, students practice with neural headsets. Two types of headset are used: the Mindwave and the Mindflex. The neural headsets visualize the brainwaves of a student in real time and so, the student can monitor the level of attention or relaxation. By playing with the headsets, the students are challenged to smoothly rotate between a state of focused and defocused attention—a process that is vital for creativity and a skill that can be learned [53].

Each of the training sessions starts with a warming-up: A short video clip that is not aimed at developing creativity, but at making students wonder. The warming-up prepares the mind for the theory and training provided.

### Creativity measures

Seven creativity tasks were employed to measure students’ divergent thinking, convergent thinking and creative problem solving skills. Creative performance was measured at three time points (pre-measure, half-way measure, post-measure) and, therefore, three versions of each task (except the number task) were used. The task versions were counterbalanced across participants and time points. Importantly, the creativity measures differed from the trained exercises.

#### Divergent thinking

*Alternative Uses Task (AUT)*. The AUT requires participants to think of as many uses of an object as possible [54]. Participants were asked to think of as many different uses for a brick (newspaper, paperclip; depending on the task version) within 3 min. After data collection, four trained raters screened the generated ideas and eliminated incomplete or unclear ideas.

The following creativity indices were used to measure the participants’ performance on the AUT: (a) *Fluency*, the total number of ideas. (b) *Flexibility*, the total number of different categories that a participant’s ideas could be assigned to. Therefore, a pre-defined list of categories was developed based on the ideas generated by all participants. (c) *Originality*, the originality level of an idea. (d) *Creativity*, the creativity level of an idea. (e) U*sefulness*, the usefulness level of an idea. Two raters scored Originality, Creativity and Usefulness, using a 5-point scale (ranging from 1 “not at all [dimension]” to 5 “very [dimension]”). The two raters first assigned scores to a random sample of 30% of participants’ ideas, based on which the raters’ reliability (two-way random, consistency) was calculated. The results showed good intraclass coefficients (ICC) for Originality (.893), Creativity (.898), and Usefulness (.822). Hereafter, the two raters worked independently—each rater assigned scores to 50% of the remaining ideas. For each participant, a mean score of Originality, Creativity or Usefulness was calculated across all his/her ideas.

*Visual imagination task*. Participants were presented with a picture of randomly combined shapes, and they were asked to think of as many ideas as possible of what the randomly combined shapes could represent within 3 min. Participants’ performance was measured using three indices: Fluency, Flexibility and Originality. For a detailed description of these indices, see AUT above. The intraclass coefficient (ICC) for Originality was good (.811).

#### Convergent thinking

*Remote Associates Test (RAT)*. In the RAT, a task originally developed by Mednick [55], participants were presented with six sets of three cue words, and they were asked to think of a fourth word that associates with each of the three given words. For example, for the three-word set “bar, dress, glass”, the solution word is ‘cocktail’ (cocktail bar, cocktail dress, cocktail glass). Participants had 3 min to come up with answers, and an overall RAT performance was calculated (i.e., number of correct solutions).

*Convergent visual imagination task*. This task required participants to rearrange a set of coins (e.g., arranged in a triangle) into a new shape with limited moves. Participants’ performance was measured by whether they solved (score of 1) or did not solve (score of 0) the task within the given time of 3 min.

*Idea selection task*. In the idea selection task, participants had to rank order three pictures of business ideas from most creative to least creative within 4 min. These business ideas had been evaluated beforehand on creativity by 9 creativity experts. For each participant, a final creativity score was calculated using a weighted score: 3* top-1 idea (ranked as the most creative) + 2* top-2 idea (ranked as the second creative) + 1* top-3 idea (ranked as the third creative).

#### Creative problem solving

*Insight problems*. In the current study, insight problems were used to measure participants’ creative problem solving ability. Three different insight problems were used, and one is illustrated in more detail here, the two-string problem. Participants were confronted with the following situation: two strings are hanging from the ceiling, and they are further away from each other than an arm’s length. The question is how to hold the two strings at the same time. The solution is to first set one string in motion (i.e., like a pendulum), then hold the other one, and catch the swinging string. Participants’ performance was measured by whether they solved (score of 1) or did not solve (score of 0) the insight problem within the given time of 4 min.

*Number task*. In this task, participants were presented with a picture of a parking lot. The number of one parking space was invisible due to a parked car. The task is to figure out the number of the parking space. One can only solve the task if one turns the picture up-side down. This task had one version, and to measure incubation effects it was measured on all three measurement times (pre-measure, half-way measure and post-measure). Participants’ performance was measured by whether they solved (score of 1) or did not solve (score of 0) the task within the time limit of 2 min. If a participant already solved the number task at the pre-measure, his/her performance was not included in the analyses of subsequent measurement points.

### Demographics

Students’ age, gender, and educational background were investigated at the pre-measure.

## Results

### Divergent thinking

#### AUT

**Fig 1:**
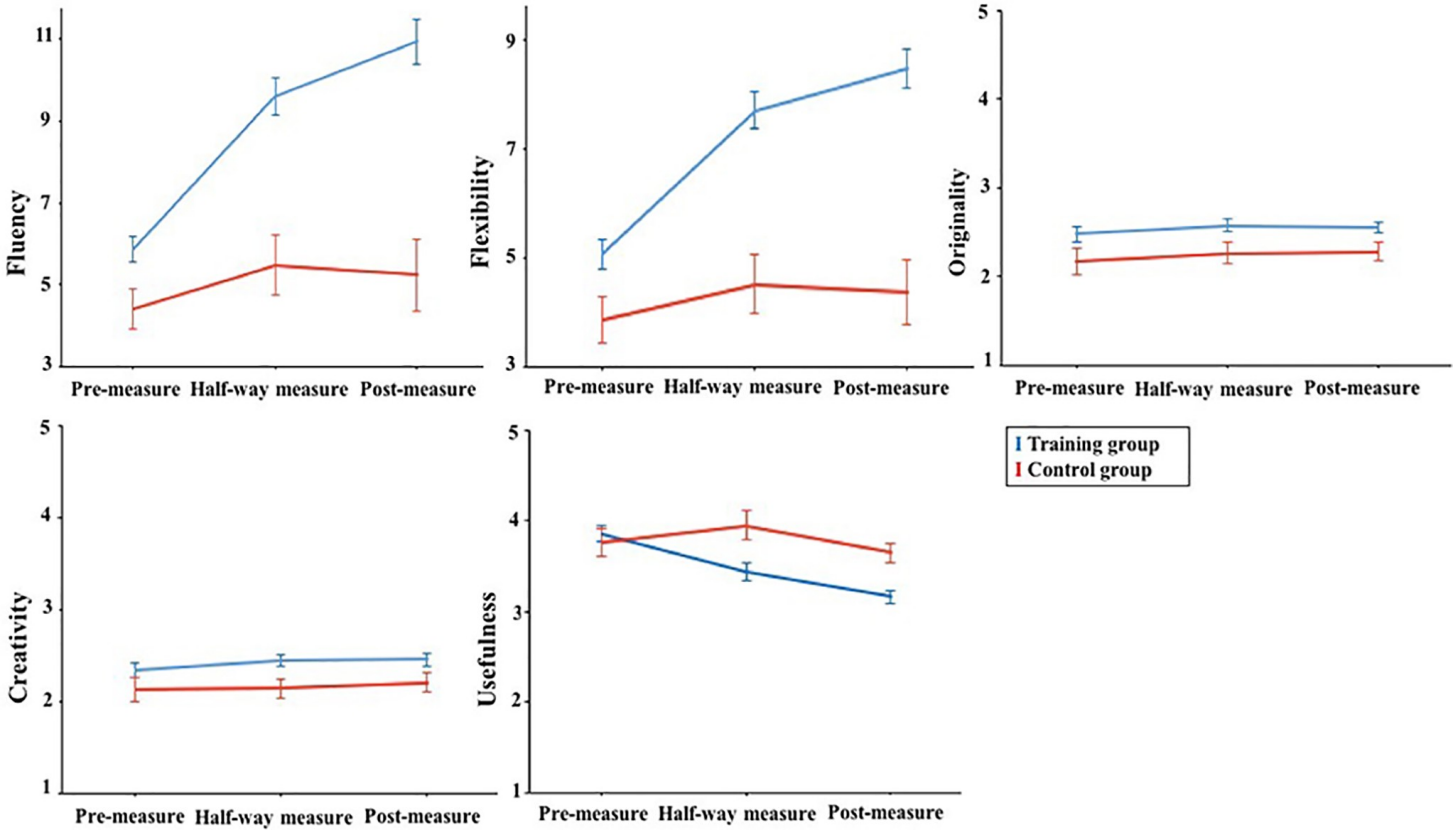
Effects of the creativity training on AUT as function of treatment and measurement time.

To examine whether the creativity training improved participants’ creative performance, we performed mixed ANOVAs in which treatment (creativity training group, control group) served as the between-subjects factor and measurement time (pre-measure, half-way measure, post-measure) as the within-subjects factor. Results are shown in Fig 1.

For Fluency, there was a significant interaction effect between training and measurement time, *F*(2,152) = 7.62, *p* = .001, η_p_^2^ = .092 (according to Cohen [56], η_p_^2^ = 0.01 refers to small effect, η_p_^2^ = 0.06 refers to medium effect, η_p_^2^ = 0.14 refers to large effect). A significant main effect was found for training, *F*(1,76) = 48.52, *p* < .001, η_p_^2^ = .393, and for measurement time, *F*(2,152) = 16.0, *p* = .006, η_p_^2^ = .095. Simple main effects (bonferroni corrected) revealed that in the training group participants’ creative performance at the pre-measure (*M* = 5.88, *SD* = 2.36) significantly differed from the half-way measure (*M* = 9.63, *SD* = 3.48, *p* < .001) and the post-measure (*M* = 10.95, *SD* = 4.49, *p* < .001), while there was no significant difference between the half-way measure and the post-measure (*p* = .141). Participants generated significantly more ideas after having followed the training, and this effect was already found after a couple of training sessions (half-way measure),and did not further increase with duration of the training (post-measure). Importantly, the control group showed no significant change from pre-measure (*M* = 4.43, *SD* = 1.83) to half-way measure (*M* = 5.48, *SD* = 2.86, *p* = .581) and post-measure (*M* = 5.24, *SD* = 2.14, *p* = 1.00), and also not from half-way measure to post-measure (*p* = 1.00).

For Flexibility, a significant interaction effect was found between treatment and time, *F*(2, 152) = 7.04, *p* = .001, η_p_^2^ = .086. A significant main effect was found for treatment, *F*(1,76) = 49.3, *p* < .001, η_p_^2^ = .397, and for measurement time, *F*(2,152) = 14.4, *p* < .001, η_p_^2^ = .161. An analysis of simple effect (bonferroni corrected) showed that the training group showed a significant improvement from the pre-measure (*M* = 5.07, *SD* = 1.98) to the half-way measure (*M* = 7.75, *SD* = 2.62, *p* < .001) and the post-measure (*M* = 8.52, *SD* = 3.02, *p* < .001). For the training group, participants improved significantly from pre-measure to half-way measure in generating ideas from different categories, but the improvement between half-way measure and post-measure was non-significant (*p* = .298). For the control group, there was no significant change neither from the pre-measure (*M* = 3.86, *SD* = 1.80) to the half-way measure (*M* = 4.52, *SD* = 2.11, *p* = .083) and the post-measure (*M* = 4.38, *SD* = 2.60, *p* = 1.00), nor from the half-way measure to the post-measure (*p* = 1.00).

For Originality, results yielded no significant interaction effect of treatment and time, *F*(2,152) = 0.023, *p* = .977, η_p_^2^ = .000. The main effect of measurement time was not significant, *F*(2,152) = 0.828, *p* = .439, η_p_^2^ = .011; but a significant main effect of treatment was revealed, *F*(1,76) = 9.49, *p* = .003, η_p_^2^ = .112; a difference between the training and control group was found regardless of measurement time.

For Creativity, the interaction effect between treatment and time was non-significant, *F*(2,152) = 0.235, *p* = .772, η_p_^2^ = .003. There was a significant main effect of training, *F*(1,76) = 7.86, *p* = .006, η_p_^2^ = .095, but the main effect of measurement time was non-significant, *F*(2,152) = 0.746, *p* = .476, η_p_^2^ = .010. The training and control group differed on creativity, but as shown in Fig 1, performance of the training group was higher on all the three measurement times as compared to the control group.

For Usefulness, there was a significant interaction effect between treatment and time, *F*(2, 152) = 3.87, *p* = .026, η_p_^2^ = .049. A significant main effect was found for training, *F*(1,76) = 8.85, *p* = .004, η_p_^2^ = .106, and for measurement time, *F*(2,152) = 6.17, *p* = .003, η_p_^2^ = .076. Interestingly, further analyses showed that performance of the training group decreased significantly from pre-measure (*M* = 3.85, *SD* = 0.632) to half-way measure (*M* = 3.46, *SD* = 0.810, *p* = .020) and post-measure (*M* = 3.16, *SD* = 0.526, *p* < .001), and from half-way measure to post-measure (*p* = .042). Though there was a decrease tendency in the training group, the average score of usefulness was still higher than the medium level (> 3). The control group showed no significant change from pre-measure (*M* = 3.76, *SD* = 0.873) to half-way measure (*M* = 3.94, *SD* = 0.582, *p* = 1.00) and post-measure (*M* = 3.65, *SD* = 0.370, *p* = 1.00), and not from half-way measure to post-measure (*p* = .377).

#### Visual imagination task

As in AUT, the training effect was analysed by means of a mixed ANOVA with treatment (creativity training group, control group) as the between-subject variable and measurement time as the within-subjects variable.

**Fig 2:**
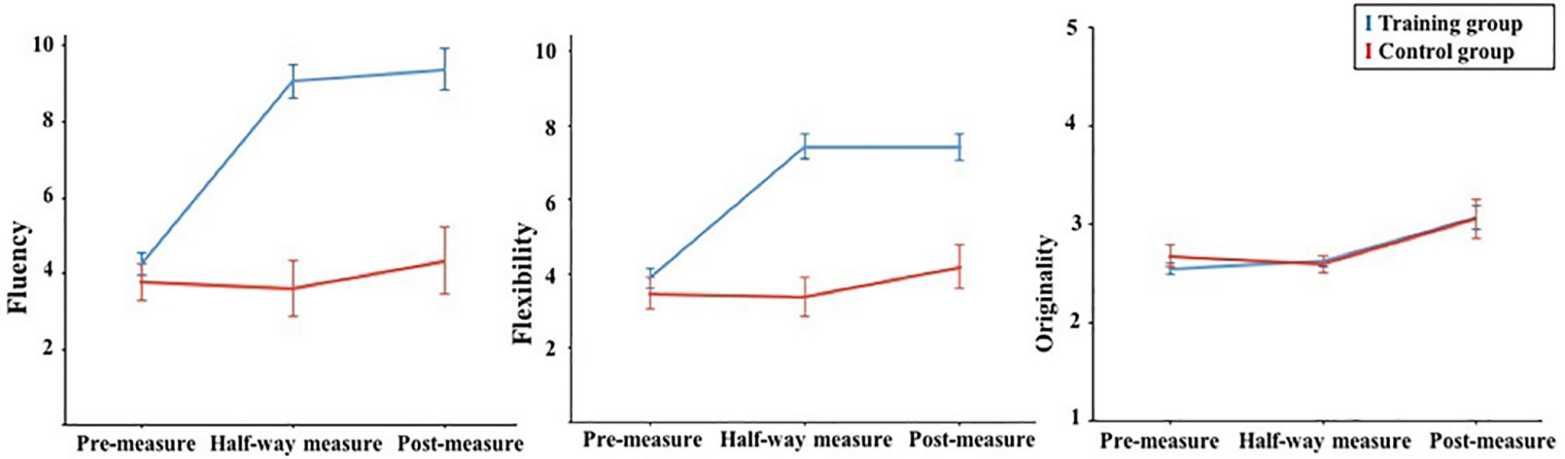
Effects of the creativity training on visual imagination task as function of treatment and measurement time.

For Fluency, the mixed ANOVA showed a significant interaction effect between treatment and time, *F*(2, 152) = 28.8, *p* < .001, η_p_^2^ = .275. A significant main effect was found for training, *F*(1,76) = 45.3, *p* < .001, η_p_^2^ = .373, and for measurement time, *F*(2,152) = 35.1, *p* < .001, η_p_^2^ = .316 (Fig 2). Simple main effect analyses using bonferroni correction indicated that in the creativity training group participants generated significantly more ideas at the half-way measure (*M* = 9.05, *SD* = 3.15, *p* < .001) and the post-measure (*M* = 9.37, *SD* = 3.83, *p* < .001) than at the pre-measure (*M* = 4.26, *SD* = 1.76), whereas no significant training effect was observed from the half-way measure to the post-measure (*p* = 1.00). For the control group, no significant change was observed from the pre-measure (*M* = 3.76, *SD* = 1.14) to the half-way measure (*M* = 3.62, *SD* = 1.28, *p* = 1.00) and the post-measure (*M* = 4.33, *SD* = 1.28, *p* = 1.00), and also not from the half-way measure to the post-measure (*p* = .755).

For Flexibility, a significance interaction effect was observed between treatment and measurement time, *F*(2, 152) = 21.4, *p* < .001, η_p_^2^ = .219. There was a significant main effect of training, *F*(1,76) = 39.3, *p* < .001, η_p_^2^ = .341, and of measurement time, *F*(2, 152) = 29.8, *p* < .001, η_p_^2^ = .282. The training significantly increased participants’ performance from the pre-measure (*M* = 3.89, *SD* = 1.59) to the half-way measure (*M* = 7.44, *SD* = 2.59, *p* < .001) and the post-measure (*M* = 7.20, *SD* = 2.57, *p* < .001); no difference was found between the half-way measure and the post-measure (*p* = 1.00). For the control group, no significant difference was found between the pre-measure (*M* = 3.48, *SD* = 1.21) and the half-way measure (*M* = 3.38, *SD* = 1.28, *p* = 1.00) and the post-measure (*M* = 4.19, *SD* = 1.33, *p* = .495); moreover, no difference was found between the half-way measure and the post-measure (*p* = .333).

For Originality, the interaction effect of treatment and measurement time was not significant, *F*(2, 152) = 0.306, *p* = .737, η_p_^2^ = .004, indicating that the training didn’t lead to an increase in the originality of the ideas generated.

### Convergent thinking

#### RAT

**Fig 3:**
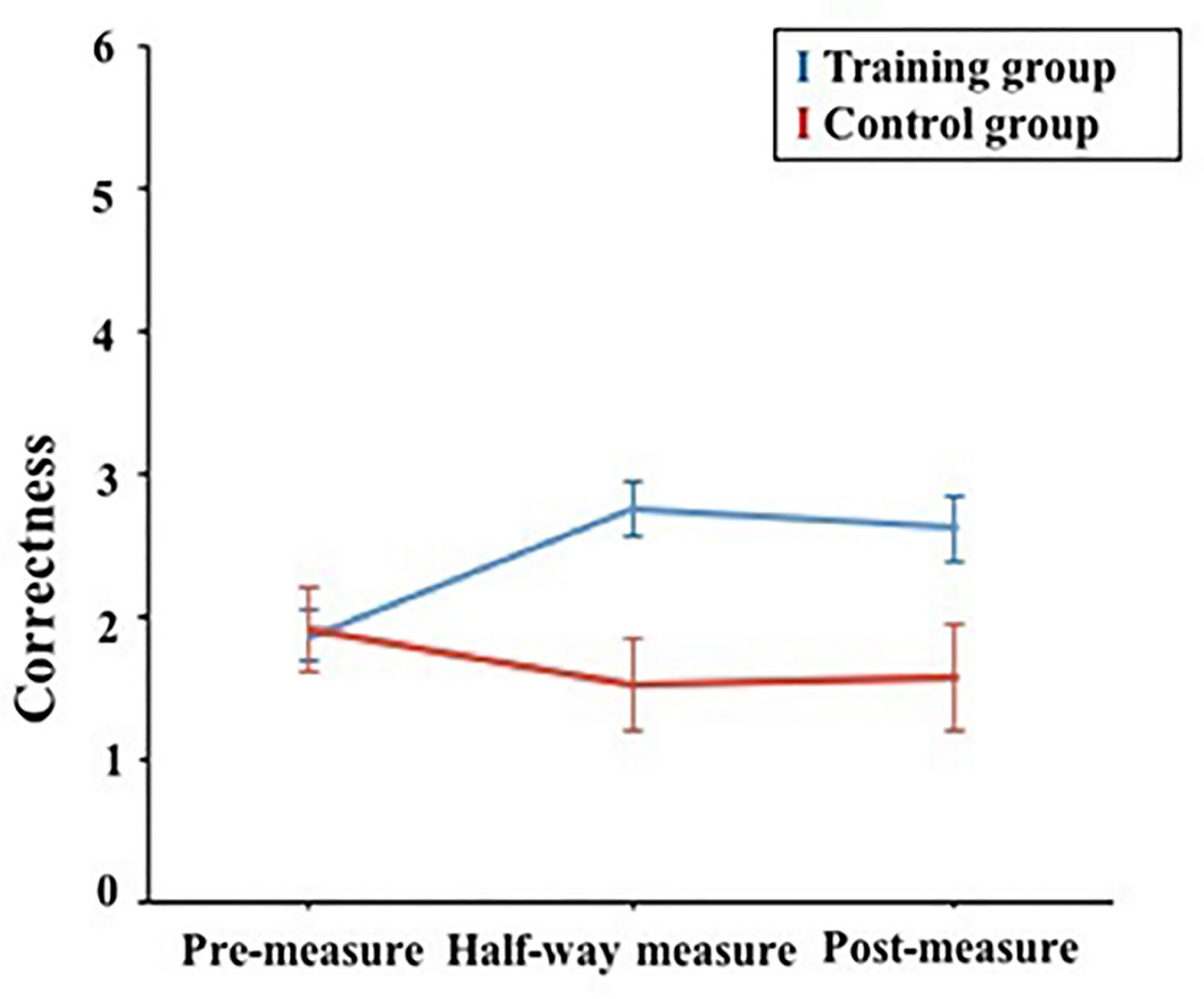
Effects of the creativity training on RAT as function of treatment and measurement time.

Using a mixed ANOVA, there was a significant interaction effect between treatment and measurement time, *F*(2, 152) = 3.55, *p* = .031, η_p_^2^ = .045. Moreover, results indicated a significant main effect of training, *F*(1, 76) = 9.05, *p* = .004, η_p_^2^ = .106; the main effect of measurement time was non-significant, *F*(2, 152) = 0.561, *p* = .572, η_p_^2^ = .007. For the training group, a simple main effect analysis (bonferroni corrected) revealed that the performance of the training group on the pre-measure (*M* = 1.86, *SD* = 1.37) differed significantly from the half-way measure (*M* = 2.75, *SD* = 1.46, *p* = .001) and the post-measure (*M* = 2.61, *SD* = 1.76, *p* = .023). However, there was no significant difference between the half-way measure and the post-measure (*p* = 1.00) in the training group. For the control group, participants’ performance on the pre-measure (*M* = 1.90, *SD* = 1.38) did not differ from the half-way measure (*M* = 1.52, *SD* = 1.44, *p* = 1.00) and the post-measure (*M* = 1.57, *SD* = 1.57, *p* = 1.00), and no difference was found between the half-way measure and the post-measure (*p* = 1.00) (Fig 3).

#### Convergent visual imagination task

Before data analyses, participants’ familiarity with the tasks were checked. On the pre-measure, 75 participants reported “unfamiliar” with the task; on the half-way measure, 67 participants were unfamiliar with the task; on the post-measure, there were 64 participants who reported “unfamiliar” with the task (see Table 1). For each measure, only participants who were unfamiliar with the tasks were included in the data analyses.

**Table 1:** Descriptive statistics of participants’ performance of the convergent visual imagination task at pre-measure, half-way measure and post-measure after checking familiarity.

		Pre-measure	Half-way measure	Post-measure
**Training**	Correct	28	24	29
	Incorrect	27	24	16
**Control**	Correct	6	7	9
	Incorrect	14	12	10
	Total	75	67	64

Given that there were some cells with expected value < 5, Fisher’s exact tests were performed to determine whether there were any differences between and within groups. Results indicated that there was no significant difference between the training and control group on the pre-measure, *p* = .124, the half-way measure, *p* = .432 and the post-measure, *p* = .268. For the training group, there was a significant improvement from the pre-measure to the half-way measure, *p* = .020; no difference was observed between the pre-measure and the post-measure, and the half-way measure and the post-measure, *p* = 1.00, *p* = .328, respectively. For the control group, no difference was found between the pre-measure and the half-way measure, and the pre-measure and the post-measure, *p* = .600, *p* = .608, respectively; the control group demonstrated a marginally significant improvement from the half-way measure to the post-measure, *p* = .050.

#### Idea selection task

Some participants did not complete this task; the performance of 64 participants could be analysed on the selection task. Mixed ANOVAs revealed that there was no interaction effect between treatment and measurement time, *F*(2, 124) = 0.517, *p* = .597, η_p_^2^ = .003. The main effect of training, *F*(1, 62) = 1.74, *p* = .192, η_p_^2^ = .033, and measurement time, *F*(2, 124) = 1.06, *p* = .348, η_p_^2^ = .004, were not significant.

### Creative problem solving

#### Insight problems

Before data analyses, participants’ familiarity with the insight problems were checked. At pre-measure, 63 participants reported that they were unfamiliar with the problems; at half-way measure, 69 participants reported “unfamiliar”; and at post-measure, 70 participants reported “unfamiliar” (see Table 2). At each measure, only participants who were unfamiliar with the insight problems were included in the data analyses.

**Table 2:** Descriptive statistics of participants’ performance of the insight problem at pre-measure, half-way measure and post-measure after checking familiarity.

		Pre-measure	Half-way measure	Post-measure
**Training**	Correct	5	1	14
	Incorrect	40	48	36
**Control**	Correct	1	1	0
	Incorrect	17	19	20
	Total	63	69	70

Given that there were some cells with expected value < 5, Fisher’s exact tests were performed to determine whether there were any differences between and within groups. We first compared the difference between the training and control group at each measurement time. On the pre-measure, Fisher’s exact test yielded a non-significant result, *p* = .662, indicating that there was no difference between the two groups prior to the training. On the half-way measure, the results were non-significant, *p* = .499. On the post-measure, the training group performed significantly better than the control group, *p* = .017. We also compared the difference within groups at each measurement time. For the training group, the difference between the pre-measure and half-way measure couldn’t be computed because the pre-measure data was a constant; there was no difference between the pre-measure and the post-measure, *p* = 1.00, and between half-way measure and post-measure, *p* = .273. For the control group, Fisher’s exact test couldn’t be computed because the half-way measure data was a constant.

#### Number task

On the pre-measure, Fisher’s exact tests revealed no difference between the training and the control group, *p* = .127. Because we aimed to examine whether participants could come up with the solution after an incubation period, we also administered the same task on the half-way measure and the post-measure. Only those participants who failed to solve the task on the pre-measure, that is, 45 participants, were included for further data analyses. Using Fisher’s exact test, their performance on the half-way measure and the post-measure were examined between groups. Results showed no significant difference between the training and control group on the half-way measure, *p* = .695, and on the post-measure, *p* = .190 (see Table 3 below).

**Table 3:** Descriptive statistics of the incubation effect between the training and control groups at pre-measure, half-way measure and post-measure.

		Pre-measure	Half-way measure	Post-measure
**Training**	Correct	21	25	29
	Incorrect	36	11	7
**Control**	Correct	12	5	5
	Incorrect	9	4	4
	Total	78	45	45

## Discussion

Creativity is important for innovation [57], everyday problem solving [58], and emotional health and wellbeing [57, 59]. It has been recognized that the need for people who are able to think creatively exceeds the degree to which creativity is available. Academics, business leaders, and policy makers around the world have stressed that creativity should be developed throughout the entire population [15]. Although creativity can be fostered [20], in most educational settings little attention is paid on developing students’ creative thinking skills. There is a strong need for well-developed, domain-unspecific, scientifically tested creativity trainings that can be easily implemented in educational settings.

The main goal of the current research was to establish whether a creativity-training designed to meet these requirements enhances students’ creative thinking skills. After having followed the creativity-training course provided in the current study, improvements in creativity were observed. On both divergent thinking measures (the verbal AUT, and the visual VIT) students generated significantly more ideas. This effect was already found after three months of training (i.e., on the half-way measure), and did not further increase with duration of training (i.e., on the post-measure). Importantly, the control group showed no change in the number of ideas generated during time.

In addition to looking at ideation skills, the current study also allows to examine the quality of the ideas generated. As mentioned earlier, creative ideas have to be both original and useful [9, 60, 61]. Thus, an idea without originality is merely a good but mundane solution to a problem, whereas an idea without usefulness is considered weird. Often organizations need workable ideas—then, mundane ideas (i.e., highly useful ideas) are fine. However, there are situations in which individuals or organizations are explicitly looking for novel ideas—when conventional ideas don’t work effectively, original ideas are of importance [62]. In the current study, the usefulness of the ideas was always on a satisfactory level as, for all measurement moments, the usefulness was higher than 3 on a 5-point scale. However, after following the training, it seems like as students in the training condition focused less on the usefulness of the ideas, as a significant decrease was observed—both from the pre-measure to the half-way measure, and from the half-way measure to the post-measure. Research has shown that people tend to perceive an incompatibility between the originality and the usefulness of an idea [63, 64], and that most individuals focus on ideas that are consistent with social norms and reject highly original ideas [65]. A decreased focus on usefulness can be considered a first step towards focusing on the originality of an idea. In the current study, however, this does not translate into an observed increase in idea originality—the originality of the ideas did not increase, and no difference in originality was found between the ideas generated by participants in the training and the control condition.

Besides the significant improvement in creative ideation (i.e., number of ideas generated), the cognitive-oriented training program also significantly enhanced participants’ ability to diversify the categories of the ideas they generated (i.e., cognitive flexibility) [45, 48]. Indeed, in the group of students that followed the creativity-training course, the cognitive flexibility was evidenced by a significant increase in the number of distinct idea categories generated half-way and post-training. There was no difference in the training condition between half-way and post-measure, suggesting that cognitive flexibility did not further increase with duration of the training. Importantly, the increase in cognitive flexibility that was observed in the training group was not observed in the control group. The creativity training, thus, enhanced students’ ability to break cognitive patterns and to overcome functional fixedness.

As the first challenge in moving from creativity to innovation is to recognize whether the available ideas have creative potential, we also examined whether the training has a positive effect on participants’ ability to recognize creative ideas. In the idea selection task, participants had to rank order three business ideas from most to least creative. The training had no effect on participants’ idea selection performance. The training also did not substantially affect participants’ performance on the convergent visual imagination task, the task where they had to re-arrange coins. For the training group, a significant increase in performance was observed from pre-measure to half-way measure, but this difference was not present anymore on the post-measure. A possible explanation for this inconsistent finding could be the way the convergent visual imagination task was administered. No actual coins—which would allow playing with the coins to find a solution—were provided due to practical considerations during the testing session. Instead, the convergent visual imagination task was handed out on paper, and participants had to draw the solution on paper. This slightly changed the essence of the task and, most importantly, formed a misfit with the creativity training program, in which students were used to play and experiment with real objects, hereby making problems tangible as much as possible. Participants’ convergent creativity was further examined by means of the RAT. Participants’ number of correctly solved RAT word pairs prior to training was compared with that following half-way and post-measure training. Compared to the pre-measure, improved performance was observed half-way and post-measure in the creativity training condition, but not in the control condition. The difference in the training condition between half-way and post-measure was not significant, suggesting that RAT performance did not further increase with duration of training.

With regard to creative problem solving skills, no difference was observed between the training and the control condition at the pre-measure, indicating equal creative problem solving skills between both groups at the start of the project. However, at the post-measure, a significant difference in creative problem solving skills was observed between the two groups; in the creativity training group a larger percentage of participants was able to solve the creative problem solving tasks as compared to the control group. Though, when looking at the training group, no difference between pre-measure and post-measure was observed. This indicates that we have to be cautious in drawing any firm conclusions with regard to creative problem solving skills.

### Strengths and limitations

The current research project included a between-subjects design with three creativity measurement points: pre-, half- and post-measure. In addition, a control condition has been used. This makes it possible to rule out any practice or learning effects on the creative performance measures. Importantly, the training exercises differed from the tasks that were used to test the effectiveness of the training—participants were therefore not trained to the criterion [20]. This shows that the training succeeded in enabling a transfer of creative thinking skills, specifically ideation skills and cognitive flexibility. The enhanced creative thinking style, however, did not translate into the generation of more original ideas—the originality score of participants’ ideas did not increase in the creativity training group, nor did the training group generate more creative ideas than the control condition. This finding suggests that the creativity training should be further fine-tuned to optimally benefit students’ creativity development.

Moreover, an important question for future research is to focus on the optimal duration of the creativity training. The current training was a one-year creativity training, and students’ creative performance was measured prior to, half-way, and after the training. Importantly, whereas a significant increase was observed on ideation skills and on cognitive flexibility from pre- to half-way measure, no further increase was observed from half-way to post-measure, suggesting that creative performance did not increase further with a longer duration of the training. A follow-up study with addition measurement moments during the first months of training can provide insight into the time that is needed to observe a training effect. This question is also interesting in the light of earlier studies showing a creativity training effect after a 2.5 hour of creativity training in both children and adults [21, 66].

In the current study, mainly Western adults participated. It is important to examine the effectiveness of the current training among Eastern participants and among other age groups, for example, among children and the elderly. Moreover, the domain generality of the training could be further examined. We assume that the training is applicable in various domains; in the current study, however, the effect of the training has been tested on standardized and well-validated creativity tasks, but not in different domains such as science, arts, and product development. Moreover, the standardized and well-validated creativity tasks that were employed in the current study during the pre-, half-way and post-measure were of relatively short nature, participants had a couple of minutes to solve the creativity task (e.g., 3 min for the AUT, and 3 min for the RAT). Time taken to creatively solve a problem is an important component of the Six Step Cycle; specifically, during step 4, 5 and 6 time plays a vital role. For practical reasons, the creativity assessment did not include tasks that needed a longer time to be solved. In a follow up study it would be interesting to more extensively test whether students’ ability to apply step 4, 5 and 6 of the Six Step Cycle increased from the pre-measure to the half-way and post-measure. Finally, no conclusions can be drawn about the long-term effects of the training. In the current study three measurement moments have been employed, but no follow-up data are available. In future research a follow-up measurement, for example 6 months after the training, could be administered to obtain information about potential long-term effects of the creativity training.

### Conclusion

Future generations will need to think creatively in order to thrive in our fast-changing world. This brings attention to the need to foster creativity. Education plays a central role in fostering creativity—not merely in elites, but in all learners. While the world has undergone revolutionary changes, teaching practices have not changed much: learning continues to focus primarily on rote learning, instead of stimulating creativity. The current findings demonstrate the effectiveness of a one-year training program in fostering creative thinking skills in applied university students. The current findings suggest that by spending some curriculum time on creativity development, we can contribute to preparing learners for a rapidly changing world after graduation.
